# Identification of Rice Transcription Factors Associated with Drought Tolerance Using the Ecotilling Method

**DOI:** 10.1371/journal.pone.0030765

**Published:** 2012-02-13

**Authors:** Shunwu Yu, Fengxian Liao, Feiming Wang, Weiwei Wen, Jiajia Li, Hanwei Mei, Lijun Luo

**Affiliations:** 1 Shanghai Agrobiological Gene Center, Shanghai Academy of Agricultural Sciences, Shanghai, China; 2 National Key Laboratory of Crop Genetic Improvement, Huazhong Agricultural University, Wuhan, China; University of California Davis, United States of America

## Abstract

The drought tolerance (DT) of plants is a complex quantitative trait. Under natural and artificial selection, drought tolerance represents the crop survival ability and production capacity under drought conditions (Luo, 2010). To understand the regulation mechanism of varied drought tolerance among rice genotypes, 95 diverse rice landraces or varieties were evaluated within a field screen facility based on the ‘line–source soil moisture gradient’, and their resistance varied from extremely resistant to sensitive. The method of Ecotype Targeting Induced Local Lesions in Genomes (Ecotilling) was used to analyze the diversity in the promoters of 24 transcription factor families. The bands separated by electrophoresis using Ecotilling were converted into molecular markers. STRUCTURE analysis revealed a value of K = 2, namely, the population with two subgroups (i.e., indica and japonica), which coincided very well with the UPGMA clusters (NTSYS-pc software) using distance-based analysis and InDel markers. Then the association analysis between the promoter diversity of these transcription factors and the DT index/level of each variety was performed. The results showed that three genes were associated with the DT index and that five genes were associated with the DT level. The sequences of these associated genes are complex and variable, especially at approximately 1000 bp upstream of the transcription initiation sites. The study illuminated that association analysis aimed at Ecotilling diversity of natural groups could facilitate the isolation of rice genes related to complex quantitative traits.

## Introduction

In recent years, various meteorological disasters, especially droughts, have seriously and frequently affected agricultural production and threatened food security in China. Although stress-resistant varieties have been developed by the use of conventional breeding methods, these methods require a long period of testing and evaluation and lead to be too costly. Currently, it is urgent for modern biotechnology to resolve stress-resistant breeding problems. Molecular breeding should be based on the collection of drought-tolerant (DT) cultivars all over the world and the comprehension of the drought-resistant mechanisms [1]. Therefore, unremitting efforts are made to find genes related to DT from complicated rice germplasms. Under long-term natural and artificial selection, DT rice is drought-tolerant and has a more stable yield under drought stress. Researchers would like to copy a map-based cloning strategy to identify the DT genes. However, DT is a complex trait, and a single site is not enough to sustain plant drought tolerance. Due to genetic map narrowdown and indisciplinable environment conditions, near-isogenic lines often lose their drought tolerance or become undetectable in the field [1], [2]. As a lucky gene, *DST* (*drought and salt tolerance*) could be located to the mutation site by the aid of leaf width because the gene controlled simultaneously both the drought-tolerant and leaf width traits [3]. Exciting reports have shown that association analysis and linkage mapping could identify polymorphisms of *lcyE* and *crtRB1* alleles responsible for beta-carotene levels in maize [4], [5]. Thus, association analysis is regarded as an effective tool to identify these genes containing complex traits based on natural population investigations. Although genome-wide association analysis through genome sequencing has be used for plant complex trait [6], [7], it is still very expensive to a laboratory, particularly in the developing countries.

TILLING (targeting-induced local lesions in genomes) provides a reverse genetics strategy that is cost-effective and high-throughput [8], [9]. The technology is based on screening interested regions of genes using PCR. It can quickly screen a chemical or a physically induced mutation population and analyze missense alleles, knockouts or even non-sense mutations in natural populations [10]. TILLING is applicable for a variety of experimental platforms of separating DNA fragments, such as agarose gel, sequencing gel, HPLC (high-performance liquid chromatography) and capillary electrophoresis [11]. Due to the technological diversity involved in TILLING, it is also widely used in Drosophila [12], zebrafish [13], wheat [14], maize [15], soybeans [16], lotus [17] and Arabidopsis. When TILLING is used to find variations of natural populations, it is termed Ecotilling [10]. At the **International Rice Research Institute**, this approach has been used to screen genes of interest in the rice germplasm pool and in mutants [18]. Nieto et al. firstly reported that a collection of *Cucumis* spp. was used for the implementation of Ecotilling to identify new allelic variants of *eIF4E* that control virus susceptibility [19]. High speed increasing genome data continuously expedites the use of TILLING, which is a cost-effective, high-throughput and reverse genetics method used in functional genomics research. However, determining how to use Ecotilling to effectively mine for gene functionality is still difficult.

Under drought stress, there are a large number of proteins expressed in plants, including transcription factors, which are a very important class of regulatory factors [20]. The transcription factor families, such as AP2/ERF, bZIP, NAC, HD-ZIP, MYB/MYC and NF-Y (Nuclear factor Y), can be induced by drought stress, and the alteration in expression of some of these genes could affect drought tolerance [21]–[25]. Some transcription factors have been discovered to have no relationship with DT and are only related to plant development. However, the over-expression or suppressed expression of the genes can enhance the drought tolerance of plants. For example, *AtHDG11*, an HD-START protein, is an important regulatory element in flower development, but its over-expression in a T-DNA mutant clearly enhances the drought tolerance of Arabidopsis [26]. Another example is a zinc finger gene *DST*, which controls leaf morphology; it can enhance rice drought tolerance after suppression [3]. These stories indicate that there is still a great job annotating the role of transcription factors.

As a *cis*-element, a promoter is an important component of a gene, and it has an important role in gene function research. The research about promoter research has currently become a hot topic. An important reason for this is that gene functional changes are caused by changes in the promoter. In rice and Arabidopsis, there are direct evidences to indicate that the gene spatiotemporal expression profile will also be changed, and unexpected changes in its function occur with changes in the sequence of the promoter. The aforementioned *AtHDG11* has been validated as a flower development gene, and it enhanced the drought tolerance of Arabidopsis when it was inserted in a super-expression promoter [26]. The gene *HKT1;1* has been inserted into a new position using an enhancer trap expression system and was only expressed specifically in the mature root stele. This led to a reduction in the root-to-shoot transfer of Na^+^; plants with reduced shoot Na^+^ also have increased salinity tolerance, but plants constitutively expressing HKT1;1 driven by the cauliflower mosaic virus 35S promoter accumulate high shoot Na+ and grow poorly [27]. In natural plant populations, promoter polymorphisms in different varieties were also found to determine the phenotype variation. *Xa13* is a key gene in pollen development. Promoter mutations in *Xa13* cause downregulation of expression during the host–pathogen interaction, resulting in the fully recessive *xa13*, which grants race-specific resistance against bacterial blight [28]. Screening gamma-ray mutants of *Arabidopsis thaliana* in low-phosphate medium, the pLPR1^Bay0^ allele became a null allele because a 16-bp deletion was located upstream of the LPR1 transcription start site and caused a loss of function of LPR1 [29]. Thus it could be concluded that the gene promoter polymorphisms in a natural population is a contributing factor of phenotypic variation.

Drought is one of the major agricultural disasters in China. To reduce losses in agricultural production caused by drought, we have long been engaged in breeding and theoretical research on drought resistance in rice at the Shanghai Agrobiological Gene Center. A set of rice DT germplasm has been collected and evaluated under both normal and drought stress conditions. In this study, the promoter polymorphism of twenty-four rice transcription factor families was analyzed using Ecotilling technology based on these materials. Drought-related transcription factors were identified by association analysis using the software TASSEL.

## Results

### Drought tolerance identification

Using the methods and facilities of DT evaluation based on the ‘line-source soil moisture gradient’ [30], the major agronomic traits and physiological characteristics of 95 rice varieties were investigated. Under normal and drought stress conditions, there were significant differences in the rice yield decrease among varieties of the population (shown in the Electronic supplementary material, Table S1). The drought significantly reduced the yields, and the main reason for this was the decline in biomass. The drought index of 60% of the varieties was more than 1.30 and reached a grade of 1. Among them, the yields of 23 varieties were characterized by upland rice whose yields under drought stress are higher than under normal condition. The drought level of approximately 26.3% of the varieties was 3, and 6.3% exhibited levels 5 and 7. Approximately 7.4% of the remaining varieties exhibited a DT level of 9. The results revealed that drought-resistant capacity of these varieties ranged from drought-resistant to drought-sensitive (Table 1).

**Table 1:** Basic information on the tested cultivated rice.

No	Name	Origin	DT index	DT level	No	Name	Origin	DT index	DT level
1	IR70358-145-1-1-B	Philippines	1.13	3	49	MIFOR6-2	Philippines	1.12	3
2	Wujianneitiangu	China	1.13	3	50	TKM6	Indian	3.40	1
3	KU10	Thailand	2.74	1	51	IR53236-275-1	Philippines	1.88	1
4	KU70-1	Thailand	1.43	1	52	AZUCNEA	Philippines	1.05	3
5	KU104	Thailand	1.98	1	53	C21	Philippines	1.47	1
6	T1095	India	1.43	1	54	DULAR	Indian	1.88	1
7	YASSI	Ivory Coast	1.12	3	55	SALAK	Indonesia	1.13	3
8	PATE BLANCMN3	Ivory Coast	1.69	1	56	PR325	Porto Rico	1.77	1
9	BPI 9-33	Philippines	1.09	3	57	PR403	Porto Rico	1.22	3
10	NEP HUONG	Vietnam	1.58	1	58	RIKUTO NORIN21	Japan	0.66	9
11	MONOLAYA	America	1.69	1	59	PRATAO	Brazil	2.17	1
12	COLOMBIA1	Columbia	1.45	1	60	CATETO	Brazil	1.72	1
13	DINALAG	Philippines	1.34	1	61	EMATA YIN	Myanmar	1.63	1
14	IAC25	Brazil	2.31	1	62	IR66417-18-1-1-1	Philippines	2.88	1
15	IAC47	Brazil	1.92	1	63	IGUAPE CATETO	Brazil	1.32	1
16	IAC1131	Brazil	2.11	1	64	DJAUB	Liberia	1.42	1
17	IAC5100	Brazil	1.15	3	65	UVS	South Africa	2.16	1
18	SILEWAH	Philippines	0.89	7	66	LAMBAYEQUE1	Peru	1.48	1
19	CHOKOTO14	Japan	1.51	1	67	IR30358-084-1-1	Philippines	0.71	7
20	IAC10	Brazil	1.42	1	68	TRES MESES	Brazil	1.66	1
21	IPEACO162	Brazil	1.12	3	69	PEROLA	Brazil	2.36	1
22	NORIN24	Japan	2.08	1	70	IAC9	Brazil	1.04	3
23	MRC 172-9	Philippines	1.38	1	71	DNJ171	Bengal	1.26	3
24	IRAT10	Cote d'ivoire	1.56	1	72	CTG680	Bengal	1.12	3
25	IRAT13	Cote d'ivoire	1.60	1	73	AMARELO	Hungary	0.98	5
26	AUS454	Bengal	1.38	1	74	MIGA	Brazil	3.42	1
27	IR43	Philippines	2.04	1	75	BENGUE	Brazil	1.70	1
28	IRAT106	Cote d'ivoire	0.47	9	76	BLCO.BRANCO	Brazil	1.76	1
29	MILTEX	Philippines	1.34	1	77	CARTUNA	Indonesia	0.46	9
30	IR10781-75-3-2-2	Philippines	1.26	3	78	IR57902-49-1-2-B	Philippines	0.88	7
31	IAC165	Brazil	1.51	1	79	IR68704-145-1-1-B	Philippines	2.09	1
32	MILT1444	Philippines	1.45	1	80	IR55423-01	Philippines	1.18	3
33	SINALOA A68	Mexico	1.06	3	81	Chaojihandao2-9	China	1.52	1
34	BLUE BELLE	Guyana	1.45	1	82	CU3069	Brazil	1.42	1
35	IR11248-148-3-2-3-3	Philippines	1.47	1	83	Liuhuangzhan	China Guangdong	0.79	7
36	IAC 164	Brazil	2.01	1	84	Qingsizhan1	China Guangdong	2.21	1
37	GAMA 318	Indonesia	1.06	3	85	IR65251-19-1-B	Philippines	0.78	7
38	KN96	Indonesia	1.67	1	86	CEIA64-S64-4	Philippines	1.04	3
39	KN361-1-8-6	Indonesia	1.31	1	87	Qingsizhan	China	1.18	3
40	ITA 117	Nigeria	2.31	1	88	Guisanzhan	China	2.11	1
41	CICA 4	Colombia	2.64	1	89	Shuangguizhan	China	1.54	1
42	IR5931-110-1	Philippines	2.48	1	90	Zhongerzhan	China Zejiang	1.47	1
43	IR65907-116-1-B	Philippines	1.23	3	91	PRATAO PRECOSE	Brazil	0.33	9
44	IR10198-66-2	Philippines	2.06	1	92	Huhan3	China Shanghai	2.13	1
45	IR7790-18-1-2	Philippines	1.19	3	93	Shenshuidao1	China Guangxi	0.47	9
46	IR6115-1-1-1	Philippines	1.47	3	94	Shenshuidao2	China Guangxi	0.66	9
47	IR388010	Philippines	1.65	1	95	Shenshuidao3	China Guangxi	0.54	9
48	IR2061-522-6-9	Philippines	1.20	3					

### Detecting the polymorphisms of transcription factor promoters through Ecotilling

The genome data of 24 transcription factor families were downloaded from the GRAMENE database. Based on 1000–1200 bp upstream sequences of genes, 505 pairs of primers were designed for Ecotilling detection (Table 2). PCR products of 204 primer pairs with length diversity after *CEL1* digestion between Nipponbare and testing materials were detected. A band phenotype appeared for the first time was marked as 1, and the band phenotypes emerged later were marked in turn. There were a maximum of 11 alleles in one primer pair that could be detected. Each primer pair detected an average of 3.36 alleles in these genes. There were 69 genes with 2 alleles, 52 genes with 3 alleles, 46 gene markers with 4 alleles and 23 gene markers with 5 alleles, which accounted for 34.2%, 25.7%, 22.8%, and 11.4% of the total number of polymorphic markers, respectively.

**Table 2:** Transcription factor families for Ecotilling.

Gene family	Gene number	Gene family	Gene number
Alfin-like	9	HRT	1
AP2-EREBP	155	HSF	22
ARR-B	8	LFY	1
BBR/BPC	4	LIM	6
BES1	6	MADS	20
C2C2-CO-like	17	PBF-2-like	2
C2C2-YABBY	7	PLATZ	12
CAMTA	6	Pesudo ARR-B	5
CCAAT-Dr1	1	RWP-RK	12
CCAAT-HAP2	11	S1Fa-like	2
CCAAT-HAP3	12	SBP	16
CCAAT-HAP5	21	sigma70-like	6

### Analysis of population structure

**Figure 1:**
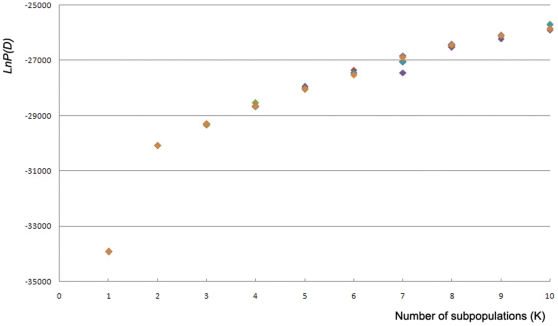
Posterior probabilities (Ln *P*(D)) from six parallel calculations for each hypothetic number of subpopulations (K) in the range of K = 1 to K = 10.

The model-based method was performed using the TILLING polymorphic loci to recognize the genetic structure of all the 95 samples based on the gene markers distributed in the whole genome. Independent calculations were performed six times for each k value from k = 1 to k = 10. The posterior probability (ln *P*(*D*)) sharply increased from k = 1 to k = 2 but slowly after k = 2 (Figure 1). The results of six parallel calculations converged with each other only for k = 1, 2, and 3. However, the clustering structures among rice accessions varied among the parallel calculations for k = 3 and k = 4. The population structure with two subpopulations (i.e., k = 2) was chosen for the samples in this study. The result was similar to the analysis of the mini-core collection of Chinese germplasms [31].

**Figure 2:**
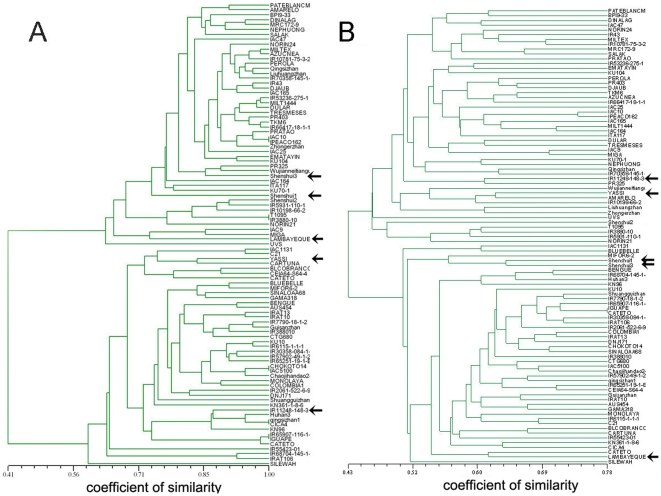
UPGMA clustering of 95 rice accessions. A, clustering based on TILLING markers of promoters; B, clustering based on InDel loci. The arrows indicate the accessions being clustered into opposite sub-populations between two trees. The horizontal axis represents the coefficient of similarity.

The population structure based on the Q values with k = 2 coincided very well with the UPGMA [32] tree from the distance-based analysis (Figure 2A). A batch of 50 accessions was grouped into one clade shown in the upper part of the dendrogram, while the other group had 45 accessions located in the lower part. Simultaneously, the population structure based on the InDel markers (Figure 2A, Table S2) also coincided well with TILLING markers, except for five accessions of the intermediate types (Figure 2).

### Association analysis between drought tolerance and polymorphisms of transcription factor promoters

The association between the DT traits and TILLING markers was calculated using a MLM function based on a simple model using TASSEL2.0 [31], and the population structure and genetic relationship among individuals were considered. The markers were distributed in 204 polymorphisms from the TILLING analysis of 24 transcription families. The significant marker-trait associations were identified by a threshold of P≤0.001, with the relative magnitude represented by the R^2^ value as the portion of variation explained by the marker. The proportion of the phenotypic variation (R^2^) shared by the marker locus variation is shown in the Electronic supplementary material, Table S3. The results of the association are shown in Table 3 when the P value was less than 0.001. The quantitative trait loci (QTL) were further searched in the GRAMENE database, and these genes all overlapped with the QTL associated with osmotic stress. Additionally, these genes also overlapped with the QTLs for grain yields that have already been reported (data not shown).

**Table 3:** The results of associated analysis between transcription factor promoters and DT traits during drought treatment.

Traits	Gene name	Gene ID	Gene family	P value	R^2^	QTL	QTL Traits	references
Drought Tolerant index	*OsALF11*	Os11g14010	Alfin-like	6.57E-04	0.20	AQAL037	biomass yield in aerobic land	[45]
	*OsGRF5*	Os02g53690	GRF	9.95E-04	0.17	AQO089	root number of upland rice	[42]
	*OsAE115*	Os07g13170	AP2/EREBP	7.88E-10	0.43	AQA047	leaf rolling	[47]
Drought Tolerant Level	*OsGRF8*	Os04g48510	GRF	3.46E-04	0.21	CQG4	osmotic tolerance	[48]
						CQAA15	leaf yellowing tolerance	[46]
	*OsNFYB12*	Os09g39490	CCAAT	1.49E-05	0.23	DQE53	relative water content	[49]
	*OsAE25*	Os02g34270	AP2/EREBP	2.12E-05	0.29	DQC5	penetrated root number of upland rice	[42]
						CQAI39	root thickness of low moisture regime	[47]
	*OsAE128*	Os08g43200	AP2/EREBP	1.27E-06	0.24	AQA010	tiller number of low moisture regime	[47]
	*OsZIM14*	Os07g42370	Tify	8.00E-04	0.13	AQAL056	deep root dry weight in aerobic land	[45]

The least significant differences (LSDs) were calculated to determine the differences among the accession groups defined by alleles or their haplotypes of associated markers (Table 4). The gene *OsAE128* marker, with two alleles, was significantly associated with the DT level trait. Accessions with allele 1 (A1) had the lower DT level and 44 accessions with A1 belong to the upper subpopulation in Figure 2 except for 4 accessions. The other 51 accessions with allele 2 (A2) had higher mean values of DT level and belong to the lower subpopulation. In addition, for the gene *OsZIM14* marker with two alleles, all accessions with A1 belong to the lower subpopulation except for the variety of DJAUB. For other gene markers, alleles distributed two subpopulations. But it is also interesting that 11 accessions with A2 in the gene *OsGRF5* were originated from Philippines and Brazil varieties.

**Table 4:** Multiple comparisons among means of accessions grouped by the alleles of associated markers.

traits	Gene names	alleles	N	mean^a^	P≤0.05	P≤0.01
DT index	*OsALF11*	A1	23	1.40	a	A
		A2	60	1.61	ac	AB
		A3	3	2.24	bcd	A
		A4	5	1.03	ad	A
		A5	4	0.82	bd	AC
	*OsGRF5*	A1	60	1.45	a	A
		A2	11	1.94	b	A
		A3	21	1.67	ab	A
		A4	3	1.40	ab	A
	*OsAE115*	A1	73	1.40	a	A
		A2	4	2.03	b	A
		A3	8	1.90	b	A
		A4	10	1.57	ab	A
DT level	*OsGRF8*	A1	16	1.63	a	A
		A2	34	2.12	a	A
		A3	27	2.41	a	A
		A4	18	4.00	b	B
	*OsNFYB12*	A1	15	3.38	a	A
		A2	41	2.74	a	A
		A3	39	1.77	b	B
	*OsAE25*	A1	35	2.31	a	A
		A2	47	2.18	a	A
		A3	4	2.00	a	A
		A4	8	4.33	b	A
	*OsAE128*	A1	44	1.76	a	A
		A2	51	3.04	b	B
	*OsZIM14*	A1	14	3.86	a	A
		A2	81	2.23	b	A

a Means followed by different letters were significantly different by the LSD test at the level P≤0.05 (in lowercases) and P≤0.01 (in uppercases).

### Sequence analysis of promoters

**Figure 3:**
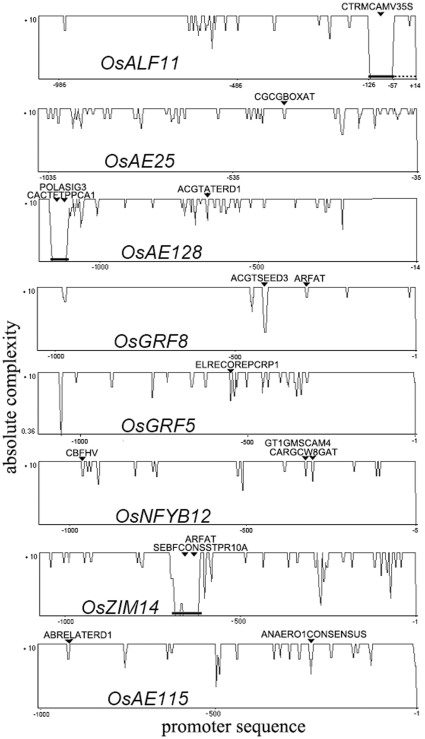
Sequence complexity of associated genes from five varieties. Black lines represent an insertion. Broken lines represent the repeat of the insertion. The arrows indicate that one or more than one site of *cis*-acting regulatory DNA elements were inserted into the promoters. The abscissa represents the distance to the transcription start sites.

The promoters of 8 genes associated with drought resistance in 5 varieties were sequenced. Regions of approximately 1 kb upstream of the transcription initiation sites were a high-incidence area of sequence polymorphisms (Figure 3). Among them, insertions of more than 50 bp occurred in the promoters of three genes (*OsALF11*, *OsAE128* and *OsZIM14* (Figure 3). There was a 58 bp repeat insertion in front of the transcription initiation site of the *OsALF11* gene in Huhan3. PLACE analysis (http://www.dna.affrc.go.jp/PLACE) revealed that the sequence polymorphism altered the plant *cis*-acting regulatory DNA elements. Compared to Shenshuidao1, a cauliflower mosaic virus 35S site (TCTCTCTCT) was appended in the insertion fragment of the promoter of the *OsALF11* gene in Huhan3. Then the site was moved to the −77 position in front of the transcription initiation site, while the site in other varieties was proximal to the transcription initiation site (Figure 3). In the *OsAE128* and *OsZIM14* genes, large fragment insertions also altered the binding sites of the transcription factors (Figure 3). Variations in *cis*-elements in associated genes from Huhan3 tended to enrich more stress-related *cis*-elements. For example, an ABRELATERD1 factor was located upstream of *OsAE115* in Huhan3, which was induced by dehydration stress and dark-induced senescence [33]. In addition, more than one ARFAT factor was appended upstream of *OsGRF8* in Huhan3, which was responsive to ABA and auxin [34]. An ELRECOREPCRP1 factor was found in the *OsGRF5* promoter of Huhan3, which was involved in pathogen- and wound-induced signaling [35]. A new CBFHV factor and a GT1GMSCAM4 factor were added to the promoter of the *OsNFYB12* gene, which was related to cold- or pathogen- and NaCl-induced expression [36], [37].

## Discussion

### Combination of Ecotilling technology with associated analysis can effectively aid in the determination of genes related to complex traits

Along with the advent of the post-genomics era and the completion of genome sequencing of several species, TILLING technology was endued with high expectations in large-scale functional genomics research [9]. However, ten years have passed, and successful examples of functional gene discovery have not been reported using the technology except for melon *eIF4E*, which was first discovered through Ecotilling in plants [19]. Therefore, relevant supporting technologies need to be developed to accelerate the application of TILLING strategy. This study focused on finding a method to link gene function with the differences determined by the TILLING technique. Association mapping has the potential of discovering gene loci responsible for multiple traits with no need to develop permanent segregating populations. In rice, five candidate genes were found to affect rice eating quality using association analysis [38]. TILLING can effectively scan the genome polymorphism. So the combination of association analysis with TILLING will make full use of TILLING function in large-scale functional genomics research. In fact, Raghavan et al. raised an agarose method instead of Li-Cor genotypers to be used in gene mapping [39]. In this study, using a simplified Ecotilling technique [40], gene sequence polymorphisms in a rice germplasm group were studied through agarose gel electrophoresis. Then the gene polymorphism was analyzed to associate the plant phenotype using the open software called TASSEL. A few transcription factors were successfully determined to be related to the DT index and DT level (Table 3). Thus as a low-cost effective method, it will shorten the time to identify the target gene and lay a solid foundation for further studies on gene function. For the same nature group, we can investigate more traits to expand the capacity of the associated analysis. In addition, we can increase the number of genes to expand the richness of gene markers. However, there are also some disadvantages with the technology. For example, it is possible that the polymorphisms of small fragments are missed in electrophoresis of simplified Ecotilling. Also, the function of the associated analysis software needs to expand to adapt more functional requirements. However, with technological advances and improvements in analysis software, the effectiveness of TILLING technology will be greatly improved.

### DT related transcription factors and rice drought tolerance

Transcription factors play important roles in plant growth, development and environmental response. In this study, only three genes were found to be associated with DT index and five genes with DT level in the 24 transcription factor families which they were from five gene families (Table 3). Of them, the Alfin-like, AP2/EREBP and CCAAT-HAP3 families altered the ability of plant resistance to osmotic stress in other species [33]–[35]. AP2/EREBP is a large family in rice, consisting of a total of 196 members (http://ricetfdb.bio.uni-potsdam.de/v3.0/). The family is not only involved in rice growth and development but also plays an important role in stress responses involving the dehydration-responsive element-binding protein and the ethylene-responsive element-binding factor [34]. The other two gene families belong to the GRF and Tify transcription factors, and these genes were thought to be only involved in plant development in previous reports [36], [37]. If the two genes from the GRF and Tify families could still promote plant normal development during drought stress, they would play an important role in maintaining the production. Among them, the *OsZIM14* gene can control the development of plant inflorescences [36]. The cDNA-AFLP analysis of upland rice also substantiated that upland rice varieties could redeploy some development elements to ensure better growth and development potential [41].

### DT associated gene loci and the location of QTLs

To adapt to the environmental changes during plant long-term evolution, the promoter region has participated in a series of changes, and some of them have caused changes in gene expression patterns. As an evolutionary effect, plants were enhanced or weakened in their ability to respond to changes in the environment [28], [29]. Some varieties had withstood a long-term directional selection and accumulated changes in some genes to improve rice drought resistance. Through Ecotilling detection, we also determined that polymorphisms in the 1 kb promoter regions in natural varieties were widespread. By association analysis of the polymorphism, eight genes were found to be significantly associated with the drought traits (Table 3). After searching the DT QTL, these genes were all co-located in the DT QTL region (Table 3). Two genes *OsGRF5* and *OsAE25* were also found at the QTL regions in a mapping population derived from a cross upland rice [42]. In addition, these genes were all located in QTLs for plant yield trait (data not shown). Because the DT indices and DT levels were calculated on the basis of grain yield per plant, the contribution of these genes to rice yield became expectable. These results suggest that this experimental method can be effectively used to find genes related to complex quantitative traits, even though the contribution rate may be relatively low. The information not only provided partial evidence of the consistency between association analysis and linkage mapping, but also encouraged the authors to take further study on the allelic diversity of these transcription factors using the diverse panel of rice germplasm.

The estimation of allelic effects showed diverse patterns for associated markers in our present study (Table 4). Allele A1 and A2 from *OsAE128* marker belong to the upper and lower subpopulation except for several accessions. In addition, A1 from *OsZIM14* marker belong to the lower subpopulation with higher DT level except for one variety. It suggested that there were not only subspecies nature but also DT nature. A2 from *OsGRF5* marker were originated from specific region. This kind of information is useful in the DT breeding program based on germplasm with distant genetic relations, by which the genetic diversity of modern varieties can be broadened.

## Materials and Methods

### Plant materials and the investigation of drought tolerance

Ninety-five domestic and foreign rice varieties were collected (Table 1). Three local deepwater rice varieties (Shenshuidao 1–3) were used as drought-sensitive controls. The field experiments were conducted twice in the field screen facility based on the ‘line-source soil moisture gradient’ [1] in the summer seasons of 2006 and 2007. All materials were sown in nurseries in late May and transplanted into two-row plots with a spacing of 20×20 cm in the field 25 days after sowing on a randomized block design with three replications. The field management and DT identification methods were performed according to a method published by Liu et al. [30]. The basic agronomic traits and grain yields were measured. The DT index was calculated by the following formula, (yield of test varieties under drought stress/yield of test varieties under normal condition)/(yield of control varieties under drought stress/yield of control varieties under normal condition). The test variety was Nipponbare. The DT level was recorded as Grade 1, 3, 5, 7 and 9 according to the DT index values, where 1 indicated a DT index ≧1.30, 3 indicated 1.00–1.29, 5 indicated 0.90–0.99, 7 indicated 0.70–0.89 and 9 indicated ≤0.69 [43].

### Promoter sequences of transcription factors and primer design

The data of the rice transcription factors were downloaded from PlnTFDB (3.0) (http://ricetfdb.bio.uni-potsdam.de/v3.0/) [44]. The sequences of the upstream 1.2 kb regions were downloaded from the GRAMENE database (http://www.gramene.org/). The primers were designed using Primer Premier Software.

### Screening polymorphisms of the promoter of transcription factors using Ecotilling

Ecotilling was conducted following Liao's protocol [40]. The PCRs were performed in 20 ul final volume with 4 ng DNA (pooled and un-pooled) and 0.5 U/reaction of Taq DNA polymerase (TaKaRa Ex Taq™). For EcoTILLING, DNA from each genotype was contrasted with Nipponbare separately in a 1∶1 ratio. The PCR and denaturation was performed as follows: one cycle at 94°C for 4 min; 30 cycles at 94°C for 30 s, 60–62°C for 30 s and 72°C for 60 s; one cycle at 72°C for 5 min; then 99°C for 10 min; 5 cycles from 95°C to 85°C with the temperature decreased by 2°C per cycle; finally 600 cycles from 85°C to 25°C with the temperature decreased by 0.1°C per cycle per second. *CEL*I extract was produced by the technique of Till et al. [15]. The digestion was carried out at 45°C for 30 min after 2 ul *CEL*I extract was added into PCR products. Then 10 ul of each digested product was resolved on a 2% agarose gel.

### Associated analysis of DT traits

The polymorphisms of the genetic population were analyzed using 30 InDels that were widely distributed across the whole genome (the Electronic supplementary material, Table S1) and were randomly selected with clear polymorphisms in the population. The primer sequence and chromosomal position of each marker were obtained from GRAMENE and NCBI databases (http://www.gramene.org; http://www.ncbi.nlm.nih.gov/). The Ecotilling electrophoresis bands were converted into molecular markers. Then the genetic structure of all 95 samples was investigated with the model-based method implemented in STRUCTURE2 (http://pritch.bsd.uchicago.edu/structure.html). The association between DT index, DT level and TILLING markers was detected based on a simple model in TASSEL2.0 (http://www.maizegenetics.net). The detailed methods were available in our previous publication [31].

### Isolation and analysis of promoter sequences

Based on the results of an associated analysis, the polymorphism markers associated with DT traits were selected for further analysis. According to band phenotype and DT level, more than 5 varieties were selected to isolate the promoter fragments that were inserted into the pGEM-T easy Vector (Promega, USA) for sequencing with a repeat. If the sequence from the repeat was not coincident, the fragment was submitted to resequence. Multiple alignments of the promoter sequences were performed using Clustal X software (http://www.clustal.org). The sequence complexity was shown using Vector NTI 8 software. The plant *cis*-acting regulatory DNA elements were located using PLACE (http://www.dna.affrc.go.jp/PLACE).

## Supporting Information

Table S1 **Grain yield and biomass per plant of 95 rice varieties under normal condition and drought condition.**
(XLSX)

Table S2 **Distribution of InDel markers on the rice chromosomes.**
(DOCX)

Table S3 **The association analysis of drought tolerant traits with the diversity of 204 transcription factor promoters.** R^2^ indicates the proportion of phenotypic variation against 204 transcription factors. P indicates the association analysis of drought tolerant traits with the diversity of 204 transcription factor promoters. The red font indicates the significantly associated genes.
(XLSX)
