# Supplementary material for: Identification of Rice Transcription Factors Associated with Drought Tolerance Using the Ecotilling Method
Source: PLoS One. 2012 Feb 13;7(2):e30765. doi: 10.1371/journal.pone.0030765 (PMC3278407; doi:10.1371/journal.pone.0030765)
Supplement: Table S2 — Distribution of InDel markers on the rice chromosomes. (DOCX) [file pone.0030765.s002.docx]

Table S2. Distribution of InDel markers on rice chromosomes.

| chromosome | InDel |
| --- | --- |
| 1 | R1M7, R1M30, R1M47 |
| 2 | R2M26, R2M37, R2M50 |
| 3 | R3M10, R3M23, R3M30, R3M53 |
| 4 | R4M17, R4M30, R4M43 |
| 5 | R5M13, R5M20, R5M30 |
| 6 | R6M14, R6M30 |
| 7 | R7M20, R7M30, R7M37 |
| 8 | R8M10, R8M23, R8M33, R8M46 |
| 9 | R9M20, R9M30, |
| 10 | R10M30, R10M40 |
| 11 | R11M23 |
